# Calcium triggers calmodulin degradation to induce EGF receptor instability and overcome non-small cell lung cancer resistance to tyrosine kinase inhibitors

**DOI:** 10.1016/j.jbc.2025.110305

**Published:** 2025-05-28

**Authors:** Luping Li, Xin Lian, Liangping Ding, Rongtian Guo, Jing Xu, Ruixiao Bai, Yong Yi, Xiangyu Li, Xiaoli Chen, Haorui Zheng, Jiexin Gao, Zhi-Xiong Jim Xiao, Mengmeng Niu

**Affiliations:** Center of Growth, Metabolism and Aging, Key Laboratory of Bio-Resource and Eco-Environment, Ministry of Education, College of Life Sciences, Sichuan University, Chengdu, China

**Keywords:** calcium, calmodulin, EGFR, FBXL2, protein degradation, NSCLC, EGFR-TKI resistance

## Abstract

Epidermal growth factor receptor (EGFR)-targeted therapy by tyrosine kinase inhibitors (TKIs) is the first-line treatment of non-small cell lung cancer (NSCLC). However, EGFR mutation-mediated TKIs resistance remains a major hurdle for NSCLC treatment. This study found the opposite roles of calcium (Ca^2+^) and its receptor calmodulin (CaM) in regulating EGFR protein stability. Elevated Ca^2+^ facilitates degradation of EGFR TKI-resistant mutant proteins, whereas CaM protects them from degradation. Mechanistically, Ca^2+^ binding to CaM triggers heat shock protein 70–mediated lysosomal degradation of CaM protein, resulting in reduced CaM binding to EGFR, thereby facilitating EGFR proteasomal degradation mediated by the E3 ubiquitin ligase FBXL2. Notably, a combination of Ca^2+^ activator curcumin, FBXL2 activator nebivolol, and osimertinib significantly inhibits EGFR^T790M/C797S^-driven TKI-resistant NSCLC growth. Together, this study highlights the importance of the negative feedback loop between Ca^2+^ and CaM and the critical regulatory role of Ca^2+^/CaM in the FBXL2-mediated EGFR degradation, providing a viable therapeutic strategy for TKI-resistant NSCLC.

Lung cancer is the most commonly diagnosed cancer and the leading cause of cancer-related death worldwide, in which non-small cell lung cancer (NSCLC) accounts for approximately 85% of all lung cancer cases (1, 2). Activating mutation of epidermal growth factor receptor (EGFR) is the primary oncogenic driver of NSCLC, with up to 50% of NSCLC patients harboring mutations in the EGFR tyrosine kinase domain (3, 4, 5). EGFR-activating mutants can sustainably stimulate downstream signaling pathways, including Ras/ERK, PI3K/AKT, and STAT, and promote tumorigenesis (6, 7, 8, 9, 10). Point mutation of Leu858 to arginine (L858R) and exon 19 deletion mutant (19 del) of EGFR are the representative EGFR mutations, accounting for approximately 90% of all EGFR mutations (11).

EGFR tyrosine kinase inhibitors (TKIs), such as erlotinib, gefitinib or osimertinib, have been approved as a first-line treatment for NSCLC patients carrying EGFR-activating mutations and have been shown to greatly improve the prognosis of NSCLC patients (12, 13, 14, 15). However, most patients eventually acquire resistance to EGFR-TKIs, with EGFR^T790M^ mutation accounting for 50% of all EGFR-acquired mutations (16, 17). Although the third-generation EGFR-TKI osimertinib has been developed to overcome EGFR^T790M^-induced the first-generation EGFR-TKI resistance, a new EGFR^C797S^ mutation has rapidly emerged to decrease the binding affinity of osimertinib to EGFR, resulting in osimertinib resistance (18). Therefore, EGFR-TKIs resistance remains a bottleneck in NSCLC treatment, and new therapeutic approaches are urgently needed to treat TKI-resistant NSCLC.

Ca^2+^ is a ubiquitous intracellular secondary messenger and a fundamental regulator of cellular physiological functions, such as cell proliferation, migration, and cell fate determination (19, 20). Dysregulation of cellular Ca^2+^ homeostasis has been shown to play a critical role in malignancy progression (21, 22). A suitable level of Ca^2+^ is required for cell growth, whereas excessive Ca^2+^ flux into the mitochondria triggers cell death (20). Overexpression of plasma membrane Ca^2+^ ATPase 2 results in low resting cytosolic Ca^2+^ to induce human epidermal growth factor receptor 2/AKT-driven breast cancer formation and growth (23). Cancer stem-like cells can escape glucose deprivation–induced apoptosis by reducing cytoplasmic Ca^2+^ levels to avoid their overload-associated cytotoxicity (24). In addition, thapsigargin, an inhibitor of the sarco/endoplasmic reticulum Ca^2+^-ATPase (SERCA), leads to a transiently increased cytoplasmic Ca^2+^ levels to inhibit tumor growth (25, 26). These findings suggest that increasing cytoplasmic Ca^2+^ levels can be an attractive new strategy for cancer treatment.

Calmodulin (CaM) is a primary Ca^2+^ sensor commonly expressed in eukaryotic cells. In response to Ca^2+^ signaling, Ca^2+^-bound CaM undergoes a significant conformational rearrangement to loosen its structure for hydrophobic interactions with a wide range of targets, including Ca^2+^-sensitive kinases, phosphatases, transcription factors, and hormone receptors (27). Ca^2+^/CaM complex has been shown to regulate EGFR phosphorylation and activation (28, 29). Nonphosphorylated Ca^2+^/CaM complex physically binds to juxtamembrane (Jx) domain of EGFR to partially activate EGFR in the presence of EGF. The semiactivated EGFR subsequently phosphorylates CaM at Y99 and Y138 sites, and EGFR can then be fully activated through p-Ca^2+^/CaM-induced phosphorylation (29). However, the effects of Ca^2+^/CaM on EGFR expression are still largely unknown. Our previous study shows that E3 ubiquitin ligase FBXL2 can promote EGFR proteasomal degradation to inhibit EGFR-TKI–resistant NSCLC growth (30). In this study, we found a negative feedback loop between Ca^2+^ and CaM and the critical role of Ca^2+^/CaM in regulation of FBXL2-mediated EGFR degradation, suggesting that targeting the Ca^2+^/CaM/FBXL2/EGFR axis may be a potential novel therapeutic strategy for the treatment of EGFR-TKI–resistant NSCLC.

## Results

### Ca^2+^ facilitates EGFR degradation, whereas CaM protects EGFR from degradation

**Figure 1:**
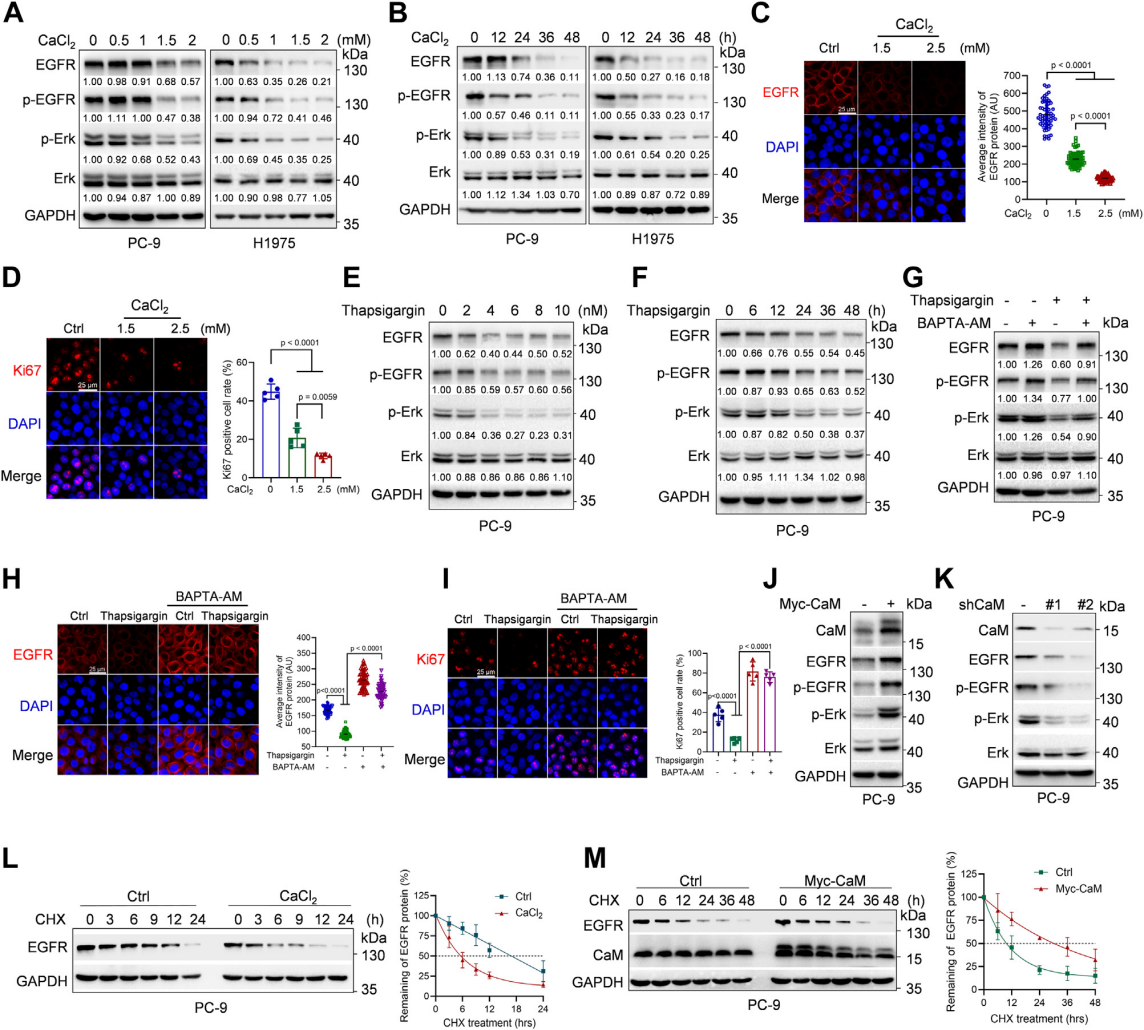
**Ca^2+^ can promote EGFR degradation, while CaM protects EGFR protein from degradation**. *A–B*, PC-9 or H1975 cells were treated with CaCl_2_ at the indicated dose for 24 h or 1.5 mM CaCl_2_ for the indicated time prior to Western blot analyses. *C*–*D*, PC-9 cells were treated with CaCl_2_ at indicated dose for 24 h, and cells were then subjected to immunofluorescence staining for EGFR or Ki67 with DAPI counterstaining (*blue*). The average intensity of EGFR protein (n = 60, each data point derived from a single cell) were presented as mean ± SD [F(2.000, 92.51) = 763.8, *p* < 0.0001, Brown-Forsythe and Welch ANOVA test followed by Dunnett’s T3 multiple comparisons test]. Ki67-positive cell rate (n = 5, each data point derived from a distinct visual field) were presented as mean ± SD [F(2, 12) = 102.3, *p* < 0.0001, one-way ANOVA followed by Tukey’s multiple comparisons test]. *E*–*F*, PC-9 cells were treated with thapsigargin at the indicated dose for 24 h or 4 nM thapsigargin for the indicated time prior to Western blot analyses. *G*–*I*, PC-9 cells were treated with 4 nM thapsigargin for 24 h or/and 20 μM BAPTA-AM for 24 h, and cells were then subjected to Western blotting (*G*) or immunofluorescence staining for EGFR or Ki67 with DAPI counterstaining (*blue*) (*H*–*I*).The average intensity of EGFR protein (n = 60, each data point derived from a single cell) were presented as mean ± SD [F(3, 236) = 1272, *p* < 0.0001, one-way ANOVA followed by Tukey’s multiple comparisons test]. Ki67-positive cell rate (n = 5, each data point derived from a distinct visual field) were presented as mean ± SD [F(3, 16) = 121.5, *p* < 0.0001, one-way ANOVA followed by Tukey’s multiple comparisons test]. *J*–*K*, PC-9 cells stably expressing CaM or silencing CaM (shCaM-#1; shCaM-#2) were subjected to Western blot analyses. *L*, PC-9 cells were treated with 1.5 mM CaCl_2_ for 24 h, and cells were then treated with 50 μg/ml CHX at indicated time prior to Western blot analyses. The EGFR protein levels were quantified, and the half-life curves were plotted. Data were presented as mean ± SD of three independent experiments. *M*, PC-9 cells stably expressing CaM or control were treated with 50 μg/ml CHX at indicated time prior to Western blot analyses. The EGFR protein levels were quantified and the half-life curves were plotted. Data were presented as mean ± SD of three independent experiments. The protein bands in western blots were quantitated and normalized to GAPDH. Ca^2+^, calcium; CaM, calmodulin; EGFR, epidermal growth factor receptor; CHX, cycloheximide.

To explore the effects of Ca^2+^ on EGFR protein expression, NSCLC cell lines PC-9 (harboring an EGFR activating mutation, EGFR exon 19 del) or H1975 (harboring a TKI-resistant mutation, EGFR^L858R/T790M^) were treated with or without CaCl_2_. As shown in Figure 1, *A* and *B*, CaCl_2_ treatment markedly downregulated EGFR expression and inhibited its downstream pathway ERK phosphorylation in a dose- or time-dependent manner. In addition, immunofluorescence staining showed that CaCl_2_ treatment significantly reduced EGFR protein levels on the plasma membrane and decreased Ki67 positive cells in a dose-dependent manner (Fig. 1, *C* and *D*). These results indicate that Ca^2+^ is a critical factor in downregulating EGFR protein expression, inhibiting EGFR downstream pathway activation, and suppressing cell proliferation. In keeping with this notion, increasing intracellular Ca^2+^ with thapsigargin, which irreversibly inhibits SERCA to eliminate endoplasmic reticulum Ca^2+^ uptake, significantly decreased EGFR levels on the plasma membrane, inhibited EGFR downstream pathway activation, and reduced Ki67-positive cells, all of which could be effectively rescued by a Ca^2+^ chelator 1,2-bis(2-aminophenoxy)ethane-N,N,N',N'-tetraacetic acid acetoxymethyl ester)(Fig. 1, *E*–*I*). These results indicate that the inhibitory effect of thapsigargin on EGFR protein expression is dependent on increased intracellular Ca^2+^ levels.

We then examined the mechanisms by which Ca^2+^ inhibits EGFR protein expression. Since it is well documented that CaM is a classical sensor for Ca^2+^, we thus explored whether CaM is involved in Ca^2+^-induced EGFR downregulation. Interestingly, we found that in opposite to Ca^2+^, ectopic expression of CaM led to increased EGFR expression levels, while silencing of CaM inhibited EGFR expression and its downstream pathway activation in PC-9 cells (Fig. 1, *J* and *K*). In keeping with these observations, CaCl_2_ treatment markedly shortened the half-life of EGFR protein, while ectopic expression of CaM prolonged the half-life of EGFR protein (Fig. 1, *L*–*M* and Fig. S1, *A*–*B*). In addition, either CaCl_2_ treatment or ectopic expression of CaM had little effect on steady-state EGFR mRNA levels (Fig. S1, *C* and *D*). These results demonstrate that Ca^2+^ and CaM have the opposite regulatory functions on EGFR protein stability.

### Ca^2+^ binding to CaM triggers a heat shock protein 70–mediated lysosomal degradation of CaM protein, thereby inhibiting EGFR expression

**Figure 2:**
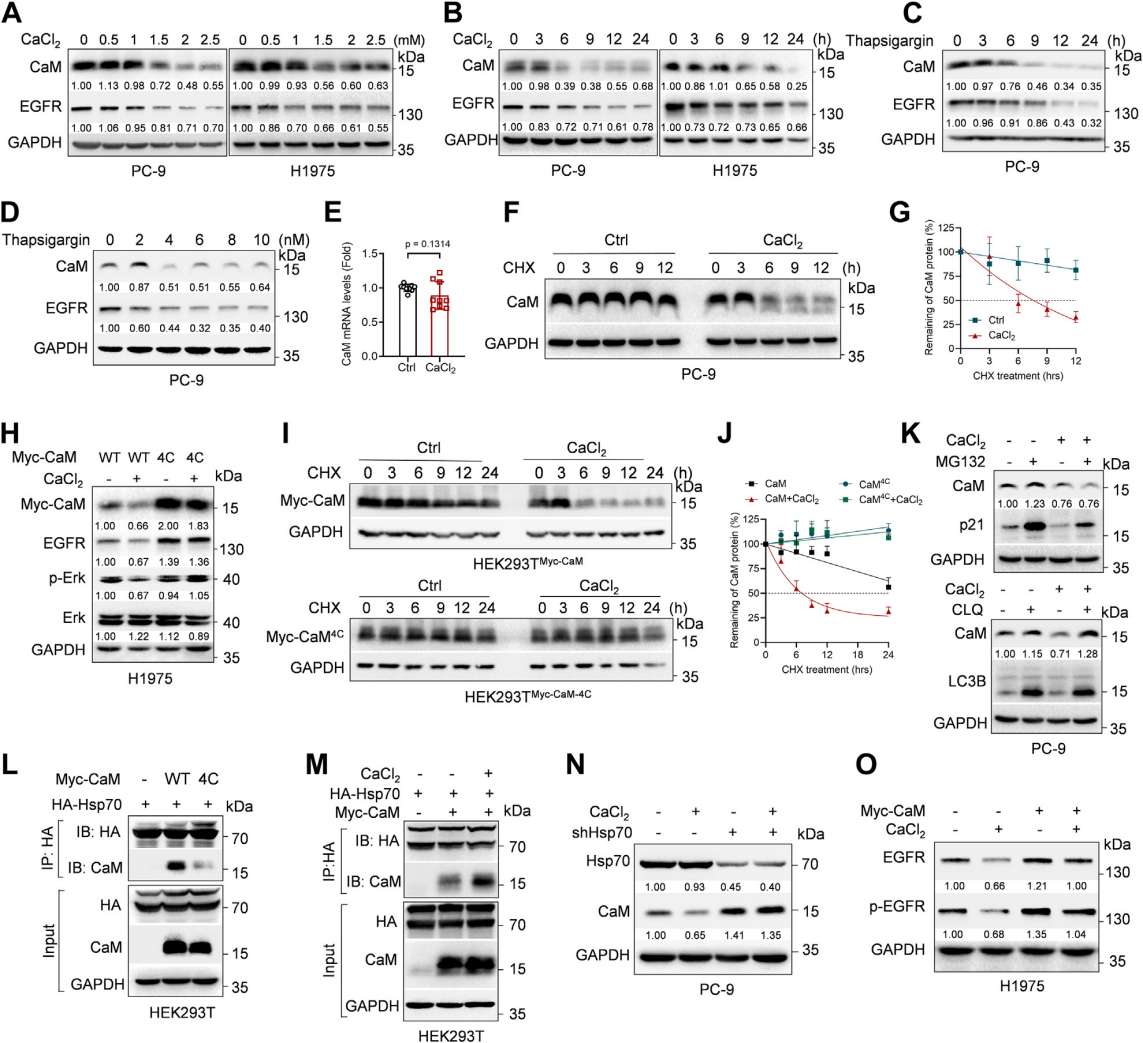
**Ca^2+^ binds to and targets CaM for lysosomal degradation, thereby inhibiting EGFR expression**. *A*–*D*, PC-9 or H1975 cells were treated with CaCl_2_ or thapsigargin at the indicated dose or time, and cells were then subjected to Western blot analyses. *E*, PC-9 cells were treated with or without 1.5 mM CaCl_2_ for 24 h prior to Q-PCR analyses. Data were presented as mean ± SD of three independent experiments performed in triplicates (t_16_ = 1.590, unpaired Student’s *t* test). *F*–*G*, PC-9 cells were treated with 1.5 mM CaCl_2_ for 12 h, and cells were then treated with 50 μg/ml CHX at indicated time prior to Western blot analyses. The CaM protein levels were quantified, and the half-life curves were plotted. Data were presented as mean ± SD of three independent experiments. *H*, H1975 cells stably expressing Myc-CaM or Myc-CaM^4C^ were treated with 1.5 mM CaCl_2_ for 24 h prior to Western blot analyses. *I*–*J*, HEK293T cells were transfected with Myc-CaM or Myc-CaM^4C^ expressing plasmids for 24 h. Cells were then treated with or without 1.5 mM CaCl_2_ for 24 h, and 50 μg/ml CHX were added at indicated time prior to Western blot analyses. The CaM protein levels were quantified, and the half-life curves were plotted. Data were presented as mean ± SD of three independent experiments. *K*, PC-9 cells were treated with 1.5 mM CaCl_2_ for 24 h or/and 20 μM MG132 for 6 h or 45 μM chloroquine for 36 h prior to Western blot analyses. *L*, HEK293T cells were cotransfected with indicated expressing plasmids, and after 12 h, cells were then treated with 45 μM chloroquine for 36 h prior to Co-IP-Western blot analyses. *M*, HEK293T cells were cotransfected with indicated expressing plasmids, and cells were then treated with or without 1.5 mM CaCl_2_ for 24 h or with 45 μM chloroquine for 36 h prior to Co-IP-western blot analyses. *N*, PC-9 stable cells were treated with or without 1.5 mM CaCl_2_ for 24 h prior to Western blot analyses. *O*, H1975 cells stably expressing Myc-CaM or control were treated with or without 1.5 mM CaCl_2_ for 24 h prior to Western blot analyses. The protein bands in Western blots were quantitated and normalized to GAPDH. Ca^2+^, calcium; CaM, calmodulin; EGFR, epidermal growth factor receptor.

It is reported that Ca^2+^ can directly bind to CaM to activate its downstream signaling pathways, yet the effect of Ca^2+^ on CaM protein expression is still unknown. Given the opposite function of Ca^2+^ and CaM on EGFR protein stability, we speculated that Ca^2+^ may inhibit CaM protein expression to suppress EGFR expression. We thus first examined the effects of Ca^2+^ on CaM protein expression. As shown in Figure 2, *A*–*D*, CaCl_2_ or thapsigargin treatment markedly inhibited CaM expression in both dose- and time-dependent manner in PC-9 or H1975 cells. In addition, CaCl_2_ treatment shortened the half-life of CaM protein, while it had little effects on steady-state mRNA levels of CaM (Figs. 2, *E*–*G* & S2*A*). Furthermore, in contrast to wildtype CaM, the expression and protein stability of CaM^4C^ mutant defective in Ca^2+^-binding ability (31) could not be regulated by CaCl_2_ treatment (Figs. 2, *H*–*J* & S2*B*). These results indicate that Ca^2+^ can promote CaM protein degradation, which is dependent on their binding.

We then deciphered the mechanism by which Ca^2+^ promotes CaM degradation. As shown in Figure 2*K*, a lysosome inhibitor chloroquine effectively rescued Ca^2+^-induced CaM degradation, while a proteasome inhibitor MG132 failed to do so, indicating that Ca^2+^ can promote CaM lysosomal degradation, but not proteasomal degradation. It has been reported that heat shock protein 70 (Hsp70) plays critical roles in protein folding, disaggregation, and lysosome-mediated degradation, we therefore examined whether Hsp70 is involved in Ca^2+^-mediated CaM degradation. As shown in Fig. 2*L*, Hsp70 could interact with wildtype CaM, in keeping with previous report (32). Of note, the interaction between Hsp70-CaM^4C^ mutant was much lesser than that of Hsp70-CaM, indicating that Ca^2+^ binding to CaM is essential for CaM interaction with Hsp70. In keeping with this notion, CaCl_2_ treatment could increase CaM-Hsp70 binding (Fig. 2*M*). Furthermore, silencing of Hsp70 could promote CaM protein expression and effectively rescued Ca^2+^-mediated CaM downregulation (Fig. 2*N*), indicating that Hsp70 is a critical downstream effector of Ca^2+^-mediated CaM degradation. Moreover, ectopic expression of CaM markedly rescued Ca^2+^-induced EGFR downregulation (Fig. 2*O*). Together, these results demonstrate that Ca^2+^ binding to CaM triggers a Hsp70-mediated lysosomal degradation of CaM protein to inhibit EGFR expression, suggesting a negative feedback loop between Ca^2+^ and CaM in maintaining cellular homeostasis by preventing overactivation of the Ca^2+^-CaM signaling pathway.

### Elevated intracellular Ca^2+^ levels inhibit CaM expression to reduce CaM binding to EGFR, thereby facilitating FBXL2-mediated EGFR degradation in an EGF-independent manner

**Figure 3:**
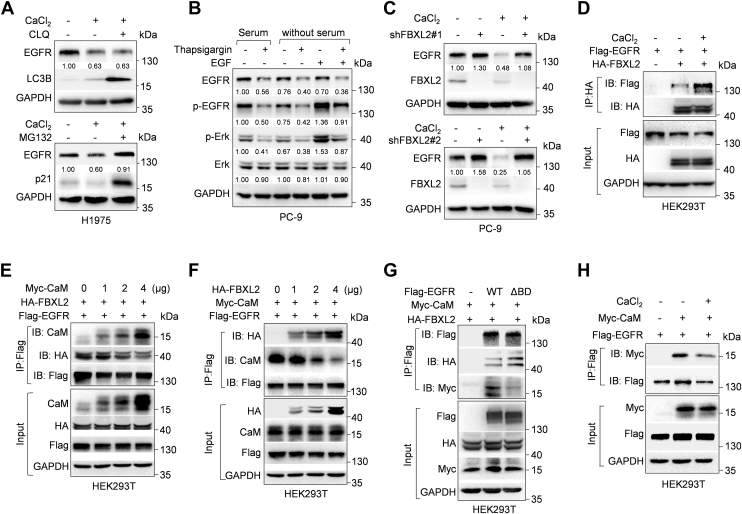
**Ca^2+^ leads to a decrease in CaM binding to EGFR and an increase in FBXL2 binding to EGFR, thus facilitating EGFR degradation**. *A*, H1975 cells were treated with 1.5 mM CaCl_2_ for 24 h, and cells were then treated with either 45 μM chloroquine for 36 h or 20 μM MG132 for 6 h prior to Western blot analyses. *B*, PC-9 cells cultured in media with or without serum were treated with 4 nM thapsigargin for 24 h or/and 100 ng/ml EGF for 15 min, and cells were then subjected to Western blot analyses. *C*, PC-9 cells stably silencing FBXL2 (shFBXL2-#1; shFBXL2-#2) or control were treated with or without 1.5 mM CaCl_2_ for 24 h prior to Western blot analyses. *D*, HEK293T cells were cotransfected with indicated expressing plasmids for 36 h before treated with or without 1.5 mM CaCl_2_ for 24 h. Cells were then treated with 20 μM MG132 for 6 h prior to Co-IP-Western blot analyses. *E*–*G*, HEK293T cells were cotransfected with indicated expressing plasmids for 36 h. Cells were then treated with 20 μM MG132 for 6 h prior to Co-IP-Western blot analyses. *H*, HEK293T cells were cotransfected with indicated expressing plasmids for 36 h before treated with or without 1.5 mM CaCl_2_ for 24 h. Cells were then treated with 20 μM MG132 for 6 h prior to Co-IP-Western blot analyses. The protein bands in Western blots were quantitated and normalized to GAPDH. Ca^2+^, calcium; CaM, calmodulin; EGFR, epidermal growth factor receptor.

We then deciphered the molecular basis by which the Ca^2+^-CaM axis regulates EGFR protein stability. As shown in Figure 3*A* and S3*A*, CaCl_2_ treatment or silencing of CaM could facilitate EGFR proteasome-mediated degradation but not lysosome-mediated degradation. Since our previous study has identified that F-box protein FBXL2 is a critical E3 ubiquitin ligase to promote EGFR proteasomal degradation in an EGF-independent manner, it is possible that FBXL2 may be involved in Ca^2+^-mediated EGFR degradation. Indeed, similar to FBXL2, Ca^2+^ led to decreased EGFR expression independently of EGF (Fig. 3*B*). Furthermore, silencing of FBXL2 effectively rescued Ca^2+^-induced EGFR degradation (Fig. 3*C*). These results demonstrate that FBXL2 is a critical downstream effector of Ca^2+^ in promoting EGFR degradation.

To decipher the mechanism(s) by which Ca^2+^ promotes FBXL2-induced EGFR degradation, we thus examined whether Ca^2+^ could affect FBXL2 expression or FBXL2 binding to EGFR. Interestingly, Ca^2+^ had little effects on FBXL2 expression, while it markedly increased FBXL2 binding to EGFR (Figs. 3*D* & S3*B*). Our previous study shows that glucose-regulated protein 94 can interact with EGFR-Jx domain to compete with FBXL2 for EGFR binding. Intriguingly, CaM has also been reported to interact with EGFR-Jx domain. We thus hypothesized that CaM may compete with FBXL2 for EGFR binding to protect EGFR from degradation. Indeed, co-immunoprecipitation experiments showed that increased CaM expression could decrease FBXL2 binding to EGFR, while increased FBXL2 expression could reduce CaM binding to EGFR (Fig. 3, *E* and *F*). In addition, EGFR mutant lacking in CaM-binding motif could bind less CaM and more FBXL2 (Fig. 3*G*). Importantly, Ca^2+^ led to a decrease in CaM binding to EGFR (Fig. 3*H*). Together, these results indicate that Ca^2+^ can promote CaM degradation, leading to decreased CaM-EGFR binding and increased FBXL2-EGFR binding, thereby facilitating FBXL2-mediated EGFR proteasomal degradation.

### Curcumin significantly inhibits EGFR-TKI–resistant NSCLC growth via inhibiting CaM expression

**Figure 4:**
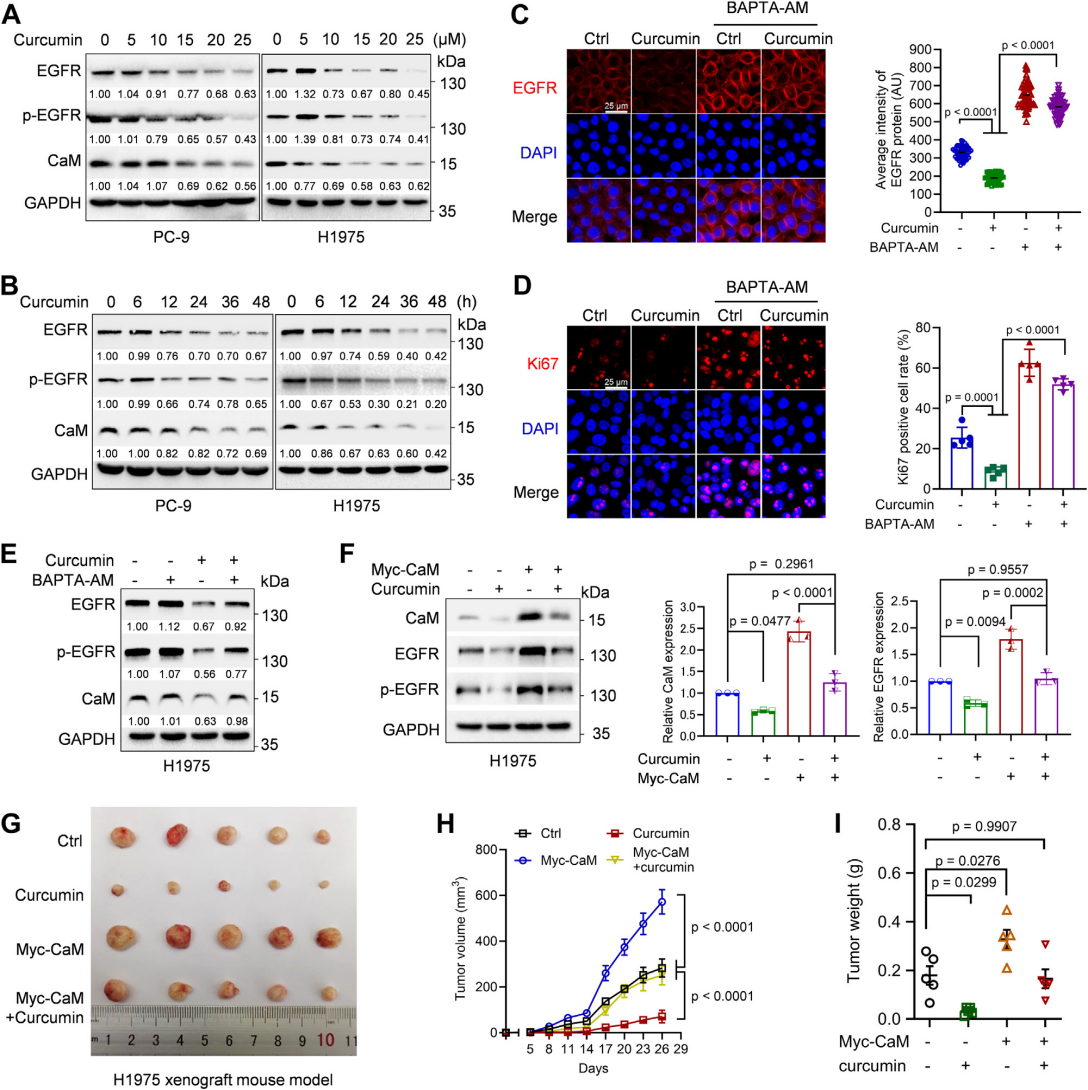
**Ca^2+^ significantly inhibits EGFR-TKI-resistant NSCLC growth in a CaM-dependent manner**. *A–B*, PC-9 or H1975 cells were treated with curcumin at the indicated dose for 24 h or 20 μM curcumin for the indicated time, and cells were then subjected to Western blot analyses. *C*–*D*, PC-9 cells were treated with 20 μM curcumin for 36 h or/and 20 μM BAPTA-AM for 24 h, and cells were then subjected to immunofluorescence staining for EGFR or Ki67 with DAPI counterstaining (*blue*). The average intensity of EGFR protein (n = 60, each data point derived from a single cell) were presented as mean ± SD (*p* < 0.0001, Kruskal*–*Wallis test followed by Dunn’s multiple comparisons test). Ki67-positive cell rate (n = 5, each data point derived from a distinct visual field) was presented as mean ± SD [F(3, 16) = 145.7, *p* < 0.0001, one-way ANOVA followed by Tukey’s multiple comparisons test]. *E*, H1975 cells were treated with 20 μM curcumin for 36 h or/and 20 μM BAPTA-AM for 24 h prior to Western blot analyses. *F*, H1975 cells stably expressing Myc-CaM or a vector control were treated with or without 20 μM curcumin for 36 h prior to Western blot analyses. The CaM and EGFR protein levels were quantified and presented as mean ± SD of three independent experiments [CaM: F(3, 8) = 74.84, *p* < 0.0001; EGFR: F(3, 8) = 57.52, *p* < 0.0001, one-way ANOVA followed by Tukey’s multiple comparisons test]. *G*–*I*, H1975 cells stably expressing Myc-CaM or a vector control were subjected to xenograft tumor growth assay (n = 5/group), and mice were treated with or without curcumin. The photos of tumor (*G*), tumor growth curves [H; time factor: F(8, 144) = 113.5, *p* < 0.0001, treatment factor: F(3, 144) = 112.5, *p* < 0.0001, and their interaction: F(24, 144) = 13.77, *p* < 0.0001, two-way ANOVA followed by uncorrected Fisher’s LSD test] and tumor weights [I; F(3, 16) = 13.24, *p* = 0.0001, one-way ANOVA followed by Tukey’s multiple comparisons test] were shown. Data were presented as mean ± SEM of five biological replicates. The protein bands in Western blots were quantitated and normalized to GAPDH. Ca^2+^, calcium; CaM, calmodulin; EGFR, epidermal growth factor receptor; TKI, tyrosine kinase inhibitor.

Given the inhibitory function of Ca^2+^ on TKI-resistant EGFR mutant expression and the fact that curcumin functions as a Ca^2+^ activator, we thus investigated the effect of increased intracellular Ca^2+^ levels induced by curcumin on the growth of EGFR-TKI–resistant NSCLC. As shown in Figure 4, *A* and *B*, curcumin markedly downregulated the expression of CaM and EGFR protein in both dose- and time-dependent manner in PC-9 and H1975 cells. Since curcumin can regulate various intracellular signaling pathways, we used a Ca^2+^ chelator BAPTA-AM to identify whether curcumin-induced EGFR downregulation was dependent on increased intracellular Ca^2+^. Indeed, as shown in Figure 4, *C*–*E*, BAPTA-AM effectively rescued curcumin-mediated either downregulation of EGFR expression on plasma membrane or inhibition of cell proliferation as indicated by Ki67-positive cells.

We further used xenograft mouse model to examine the effects of curcumin on EGFR-TKI resistant NSCLC growth. As shown in Figure 4, *F*–*I*, curcumin significantly inhibited CaM and EGFR expression, and suppressed EGFR-TKI resistant NSCLC growth in H1975 xenograft mouse model. To examine whether curcumin-induced tumor growth inhibition is dependent on CaM downregulation, we performed rescue assays. Our results showed that ectopic expression of Myc-CaM elevated EGFR protein levels and promoted H1975 xenograft tumor growth (Fig. 4, *F*–*I*). Importantly, restoration of CaM expression could effectively rescue curcumin-induced EGFR downregulation and tumor growth inhibition (Fig. 4, *F*–*I*). These results demonstrate that curcumin can suppress TKI-resistant NSCLC growth *via* CaM downregulation.

### Curcumin combined with the FBXL2 activator nebivolol significantly overcomes osimertinib resistance

**Figure 5:**
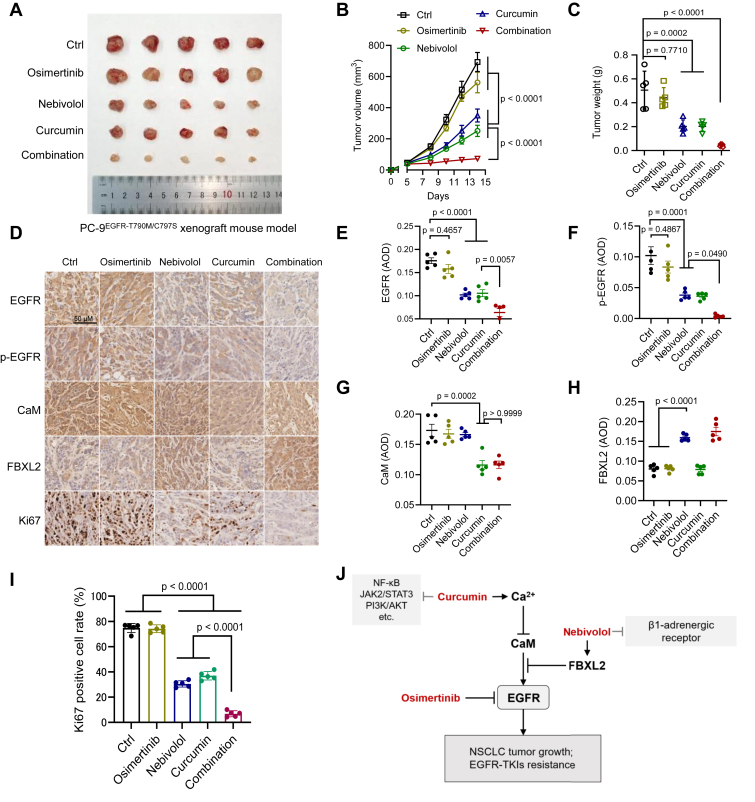
**The combination of curcumin and nebivolol significantly overcomes EGFR^T790M/C797S^-induced osimertinib resistance of NSCLC**. *A*–*I*, PC-9^EGFR-T790M/C797S^ cells were subjected to xenograft mouse model assay (n = 5/group). Mice were administrated with osimertinib, nebivolol, curcumin alone, or in a combination. The photos of tumor (*A*), tumor growth curves [B; time factor: F(5, 120) = 175.0, *p* < 0.0001, treatment factor: F(4, 120) = 90.29, *p* < 0.0001, and their interaction: F(20, 120) = 16.21, *p* < 0.0001, two-way ANOVA followed by uncorrected Fisher’s LSD test] and tumor weights [C; F(4, 20) = 24.34, *p* < 0.0001, one-way ANOVA followed by Tukey’s multiple comparisons test] were shown. The tumors were then subjected to immunohistochemistry staining for protein expression levels of EGFR, p-EGFR, CaM, FBXL2, and Ki67 (*D*–*I*). Protein expression intensity was quantified by AOD and Ki67-positive cell rate was calculated using ImageJ [EGFR: F(4, 20) = 37.74, *p* < 0.0001; p-EGFR: F(4, 20) = 24.52, *p* < 0.0001; CaM: F(4, 20) = 15.67, *p* < 0.0001; FBXL2: F(4, 20) = 59.25, *p* < 0.0001; Ki67: F(4, 20) = 470.3, *p* < 0.0001, one-way ANOVA followed by Tukey’s multiple comparisons test]. Data were presented as mean ± SEM of five biological replicates. *J*, a working model of this study. Ca^2+^ binding to CaM triggers Hsp70-mediated lysosomal degradation of CaM protein, resulting in reduced CaM-EGFR binding and thereby facilitating EGFR proteasomal degradation mediated by FBXL2. Combination of the Ca^2+^ activator curcumin and the FBXL2 activator nebivolol significantly overcomes EGFR^T790M/C797S^-mediated osimertinib resistance of NSCLC. AOD, average optical density; Ca^2+^, calcium; CaM, calmodulin; EGFR, epidermal growth factor receptor; NSCLC, non-small cell lung cancer; TKI, tyrosine kinase inhibitor.

Nebivolol, a selective β-blocker clinically used to treat cardiovascular diseases, is reported to exhibit antitumor properties by targeting the β1-adrenergic receptor (33). Our previous study has shown that nebivolol can activate FBXL2 expression to promote the degradation of EGFR-TKI–resistant mutants (30). We therefore evaluated the therapeutic potential of the Ca^2+^ activator curcumin combined with the FBXL2 activator nebivolol in the treatment of the third-generation TKI osimertinib-resistant NSCLC. As shown in Figure S4, *A*–*C*, while curcumin or nebivolol alone inhibited PC-9^EGFR-T790M/C797S^-driven NSCLC tumor growth, in consistent with previous reports that nebivolol can inhibit β1 adrenergic receptors or EGFR to suppress tumor growth (30, 33), the combination of curcumin and nebivolol exhibited significantly better inhibitory effects. Given that facilitating EGFR degradation is a potential strategy to overcome EGFR-TKI resistance (30, 34, 35, 36), we subsequently investigated the effect of nebivolol combined with curcumin on osimertinib resistance of NSCLC. As shown in Figure 5, *A*–*C*, PC-9^EGFR-T790M/C797S^-driven tumors were resistant to osimertinib, as expected. Of note, curcumin combined with nebivolol significantly overcame osimertinib resistance in the PC-9^EGFR-T790M/C797S^ xenograft mouse model. In addition, the immunohistochemistry (IHC) staining assay showed that a combination of curcumin, nebivolol, and osimertinib markedly inhibited the expression of EGFR, p-EGFR, and CaM protein and decreased Ki67-positive cells (Fig. 5, *D*–*I*). These results indicate that the combination of curcumin, nebivolol, and osimertinib may be a putative therapeutic strategy for TKI-resistant NSCLC.

## Discussion

CaM is a crucial Ca^2+^ sensor, the structure and functions of which are highly dependent on Ca^2+^ concentration (27). In response to Ca^2+^ signaling, Ca^2+^-bound CaM interacts with various targets involved in diverse important physiological activities and pathological functions, including cell cycle regulation, immune response, cardiac contraction, electrolytic balance, tumorigenesis, and tumor progression (37, 38). However, whether Ca^2+^ can regulate CaM expression is still unknown. In this study, we uncover that elevated intracellular Ca^2+^ levels trigger a lysosome-dependent degradation of CaM in a Ca^2+^-binding dependent manner, suggesting a negative feedback loop between Ca^2+^ and CaM that may play crucial roles in maintaining cellular homeostasis to prevent overactivation of the Ca^2+^-CaM signaling pathway.

The function of CaM protein can be modified by a variety of posttranslational modifications, such as phosphorylation, sulfoxidation, nitration, carboxylmethylation, and acetylation (27). Under oxidative stress, oxidized CaM protein can be degraded by heat shock protein 90 *via* 20S proteasome (39, 40). In addition, E3 ubiquitin ligase Asr1p has been reported to promote CaM monoubiquitination in a Ca^2+^-dependent manner in *Saccharomyces cerevisiae* (41). However, the molecular mechanism(s) by which CaM is degraded by the lysosomal pathway remains uncovered. The Hsp70 is a conserved and inducible protein, playing critical roles in protein folding, disaggregation, and lysosome-mediated degradation (42, 43, 44). In addition, it has been reported that Hsp70 can interact with CaM during the S phase to regulate cell-cycle progression and cell apoptosis (32). In this study, we have identified that Hsp70 is a critical factor to promote CaM lysosome-mediated degradation and that elevated intracellular Ca^2+^ levels lead to increased CaM-Hsp70 binding, thus facilitating CaM protein degradation. Our findings illuminate the key role of Hsp70 in Ca^2+^-mediated lysosomal degradation of CaM protein.

The stability of EGFR protein is tightly regulated, in particular EGFR polyubiquitination degradation mediated by E3 ubiquitin ligases or deubiquitinating enzymes. E3 ubiquitin ligase c-Cbl has been reported to bind phosphorylated Ser1045 site of EGFR to promote its endocytosis and lysosomal degradation in an EGF-dependent manner (45). E3 ubiquitin ligase CGRRF1 can bind to EGFR and promote the K48-linked ubiquitination-mediated degradation of EGFR (46). In addition, USP2a and Cezanne-1 function as deubiquitinating enzymes of EGFR to remove the ubiquitination of EGFR and protect it from degradation (47, 48). Our previous study demonstrates that the F-box protein FBXL2 binds to the kinase domain of EGFR and targets EGFR for polyubiquitin-proteasomal degradation in an EGF-independent manner (30). Of note, the roles of Ca^2+^-CaM signaling in the regulation of EGFR protein stability appear to be complex. CaM-dependent protein kinase II has been reported to promote EGFR degradation and inhibit EGFR tyrosine kinase activity (28), while calcineurin, a Ca^2+^/CaM-activated serine/threonine phosphatase, can enhance EGFR protein stability through dephosphorylation of EGFR at serine 1046/1047 and inhibiting EGFR lysosomal degradation (49). These different outcomes could be context-dependent. In this study, we have identified Ca^2+^/CaM as a novel regulator of FBXL2-mediated EGFR proteasomal degradation process. Our results show that CaM can compete with FBXL2 for EGFR binding to protect EGFR from proteasomal degradation. Importantly, increased intracellular Ca^2+^ levels can trigger CaM degradation, leading to decreased CaM-EGFR binding and increased FBXL2-EGFR binding, thereby facilitating FBXL2-mediated EGFR degradation in an EGF-independent manner. These results reveal a critical role of Ca^2+^/CaM in the regulation of FBXL2-mediated EGFR degradation.

Curcumin, a natural active polyphenol extracted from *Curcuma longa* L., is reported to increase cytosolic Ca^2+^ levels by potently inhibiting SERCA activity to prevent Ca^2+^ uptake from the cytoplasm as well as activating the Wnt-5a-mediated Ca^2+^ release to the cytoplasm (50, 51, 52, 53). It is noteworthy that curcumin, as a food component, has a variety of biological activities and a good safety profile, conferring it an attractive therapeutic adjuvant for treating disorders, including cancer. In practice, results acquired from clinical trials indeed suggest the effectiveness of curcumin in the treatment of breast cancer and multiple myeloma (54, 55, 56). Extensive mechanism studies have shown that curcumin can regulate various intracellular signaling pathways essential for cancer cell survival, growth, and invasion, such as NF-κB, JAK2/STAT3, and PI3K/AKT signalings, which underlies its broad tumor-suppressing effects against multiple cancer types (57, 58). In addition, curcumin can inhibit EGFR transcription levels and overcome gefitinib resistance by suppressing Sp1/HDAC1 binding-induced EGFR transcription activity (59). In this study, we found that curcumin can regulate EGFR protein stability in a Ca^2+^/CaM-dependent manner. In addition, we show that curcumin can significantly inhibit tumor growth *via* CaM downregulation and that combining curcumin with FBXL2 activator nebivolol markedly inhibits EGFR^T790M/C797S^ expression and overcomes EGFR^T790M/C797S^-mediated osimertinib resistance in xenograft mouse model. These results demonstrate that the Ca^2+^/CaM-EGFR axis is a crucial player in curcumin-mediated tumor growth inhibition and overcoming EGFR-TKIs resistance. In summary, our results highlight the critical role of Ca^2+^/CaM in regulation of FBXL2-mediated EGFR instability and reveal that targeting Ca^2+^ by curcumin is a potential therapeutic strategy for the treatment of EGFR-driven disorders, such like EGFR-TKI–resistant NSCLC.

## Experimental procedures

### Cell culture

PC-9 and H1975 cells were cultured in RPMI-1640 medium (Gibco) supplemented with 10% fetal bovine serum and penicillin (100 U/ml)/streptomycin (100 μg/ml). HEK293T cells were maintained in Dulbecco's modified Eagle's medium (Gibco) supplemented with 10% fetal bovine serum and penicillin (100 U/ml)/streptomycin (100 μg/ml). PC-9, H1975, and HEK293T cells were purchased from BeNa Culture Collection. Cells were cultured in a humidified incubator at 37 °C with 5% CO_2_.

### Drug treatment

MG132 (S2619), curcumin (S1848), osimertinib (AZD9291) (S7297), nebivolol hydrochloride (S1549), and BAPTA-AM (S7534) were purchased from Selleck. Chloroquine diphosphate salt (C6628) was purchased from Sigma-Aldrich. Recombinant Human EGF (236-EG) was purchased from R&D systems.

### Plasmids and shRNAs

Short hairpin RNAs (shRNAs) targeting human FBXL2, CaM, and Hsp70 were generated by inserting specific oligos into pLKO.1-puromycin lentiviral vector. The shFBXL2#1 sequences are as follows: forward (F): CCGGGCACAGATTTCCAAGGCTTTTCTCGAGAAAAGCCTTGGAAATCTGTGCTTTTTG; reverse (R): AATTCAAAAAGCACAGATTTCCAAGGCTTTTCTCGAGAAAAGCCTTGGAAATCTGTGC. The shFBXL2#2 sequences are as follows: forward (F): CCGGGCCTTTCGGGTTGCAGCAATTCTCGAGAATTGCTGCAACCCGAAAGGCTTTTTG; reverse (R): AATTCAAAAAGCCTTTCGGGTTGCAGCAATTCTCGAGAATTGCTGCAACCCGAAAGGC. The shCaM#1 sequences are as follows: forward (F): CCGGCCAGGTCAATTATGAAGAGTTTCAAGAGAACTCTTCATAATTGACCTGGTTTTTG; reverse (R): AATTCAAAAACCAGGTCAATTATGAAGAGTTCTCTTGAAACTCTTCATAATTGACCTGG. The shCaM#2 sequences are as follows: forward (F): CCGGGACAAACCTTGGAGAGAATTTTCAAGAGAAATTCTCTCCAAGGTTTGTCTTTTTG; reverse (R): AATTCAAAAAGACAAACCTTGGAGAGAATTTCTCTTGAAAATTCTCTCCAAGGTTTGTC. The shHsp70#1 sequences are as follows: forward (F): CCGGGCCATGACGAAAGACAACAATTTCAAGAGAATTGTTGTCTTTCGTCATGGCTTTTTG; reverse (R): AATTCAAAAAGCCATGACGAAAGACAACAATTCTCTTGAAATTGTTGTCTTTCGTCATGGC. The shHsp70#2 sequences are as follows: forward (F): CCGGGGACAAGTGTCAAGAGGTCATTTCAAGAGAATGACCTCTTGACACTTGTCCTTTTTG; reverse (R): AATTCAAAAAGGACAAGTGTCAAGAGGTCATTCTCTTGAAATGACCTCTTGACACTTGTCC.

Recombinant lentiviruses expressing FBXL2, wildtype EGFR, EGFR^T790M/C797S^, EGFR^ΔBD^, CaM, or CaM^4C^ (Q42C/K76C/I86C/L113C) were generated using pLVX-puro vector and amplified in HEK293T cells. The expressing plasmids of protein mutants were generated by KOD-Plus-Mutagenesis kit (Cat No. SMK-101, TOYOBO). All constructs were confirmed by direct DNA sequencing.

### Western blot analysis and co-immunoprecipitation assay

For Western blot analysis, cells were collected and lysed using EBC250 buffer (25 mM Tris-HCl, 250 mM NaCl, 0.5% Nonidet P-40, and 50 mM NaF, pH 7.4) supplemented with protease inhibitor cocktail. Equal amounts of protein samples were subjected to SDS-PAGE fractionation and transferred into PVDF membrane. Membranes were then blocked in 4% nonfat milk and incubated with primary antibody overnight at 4 °C. HRP-conjugated secondary antibody and chemiluminescence (Bio-Rad) were used for visualization of protein bands. The images were captured and analyzed using Image Lab Software 3.0. The following antibodies were used in Western blot analysis: antibodies specific for EGFR (4267), p-EGFR (3777), Erk (9102), p-Erk (9101), p21 (2947), LC3B (2775), Flag (14793), HA (3724), or Myc (2278) were purchased from Cell Signaling Technology. Antibody specific for FBXL2 (ab153842) was purchased from Abcam. Antibodies specific for glyceraldehyde-3-phosphate dehydrogenase (GAPDH, AB0037) and CaM (CY5234) were purchased from Abways Technology. Goat anti-rabbit IgG-HRP (sc-2004) antibody was purchased from Santa Cruz Biotechnology.

For co-immunoprecipitation assay, cells were lysed using lysis buffer (20 mM Tris-HCl, 125 mM NaCl, 5 mM MgCl_2_, 0.2 mM EDTA, 12% Glycerol, and 0.25% Nonidet P-40, pH 7.8). Equal amounts of total protein were incubated overnight at 4 °C with anti-HA beads or anti-Flag beads. The beads were collected by centrifugation (500 g for 30 s), washed with 0.1% NP-40 wash buffer (20 mM Tris-HCl, 125 mM NaCl, 5 mM MgCl_2_, 0.2 mM EDTA, 0.1% NP-40, pH 7.8), and then subjected to Western blot analysis. Anti-Flag M2 affinity gel (A2220) was purchased from Sigma-Aldrich. Pierce Anti-HA magnetic beads (88836) were purchased from Thermo Fisher Scientific.

### Immunofluorescence staining

Cells were fixed with 4% paraformaldehyde for 15 min, permeabilized with 0.1% Triton for 10 min, and blocked with 4% bovine serum albumin for 1 h. Cells were then stained with a specific primary antibody followed by secondary antibody. The nuclei were counterstained with DAPI. Images were acquired using a confocal fluorescence microscope. The EGFR protein levels on the plasma membrane were quantified using LAS_X software, and the Ki67 positivity rate in the nuclei was calculated using ImageJ software. The following antibodies were used in immunofluorescence staining: antibodies specific for EGFR (4267) and Ki67 (9027), as well as DAPI (8961) were purchased from Cell Signaling Technology. Rhodamine (TRITC)-conjugated AffiniPure Donkey Anti-Rabbit IgG (711–025–152, 1:160) was purchased from Jackson Immuno Research.

### Quantitative RT-PCR

Total RNAs were extracted from cells using NucleoSpin RNA plus (Macherey-nagel) and reverse transcribed into cDNA using ReverTra Ace qPCR RT Master Mix with gDNA Remover (TOYOBO) according to the manufacturers’ instructions. Q-PCR reactions were performed in a CFX-96 Real-Time PCR system (Bio-Rad). Relative quantitative values were calculated using the ΔΔCt method. The following primers were used for quantitative RT-PCR: For CaM, forward: ATGGCTGACCAACTGACTGA; reverse: CAGTTCCCAATTCCTTTGTTG. For EGFR, forward: GGCAGGAGTCATGGGAGAA; reverse: GCGATGGACGGGATCTTAG. For GAPDH, forward: GGGGAGCCAAAAGGGTCATCATCT; reverse: GAGGGGCCATCCACAGTCTTCT.

### Xenograft mouse model

H1975 (5 × 10^5^) or PC-9 (2 × 10^6^) cells were subcutaneously injected into the right flanks of 5-week-old female BALB/c nude mice (n = 5 per group). Mice were administrated with curcumin (150 mg/kg in PBS containing 5% DMSO, 40% PEG400, and 5% Tween 80 by intraperitoneal injection once daily), nebivolol (10 mg/kg in PBS containing 10% DMSO, 40% PEG400 and 5% Tween 80 by intraperitoneal injection once daily), or osimertinib (5 mg/kg in PBS containing 5% DMSO, 40% PEG400 and 5% Tween 80 by oral gavage every 3 days) alone or in combination. Tumor sizes were measured every 3 days using a caliper. At the end point, the mice were sacrificed, and the tumors were dissected and photographed. Tumor weights and volumes (calculated using formula: (length × width^2^)/2) were presented as mean ± SEM. All animal care and animal experiments were performed in accordance with the institutional ethical guidelines and were approved by the institutional review board of Sichuan University.

### Immunohistochemistry staining

Paraffin-embedded tumor samples were sectioned at a thickness of 4 μM and subjected to IHC staining. The nuclei were counterstained with hematoxylin. The sections were then scanned and imaged using Hamamatsu NDP.view2 software. The average optical density was analyzed using Image-Pro Plus 6.0. The following antibodies were used in IHC staining: antibodies specific for EGFR (4267, 1:100), p-EGFR (3777,1:100), and Ki67 (9027, 1:200) were purchased from Cell Signaling Technology. Antibodies specific for CaM (CY5234, 1:200) and FBXL2 (ab153842, 1:100) were purchased from Abways Technology and Abcam, respectively.

### Statistical analysis

The relevant experimental data were analyzed using Excel or GraphPad Prism (GraphPad Software Inc.). All data *in vitro* were expressed as mean ± SD, and all data from mouse models were expressed as mean ± SEM. Two-tailed Student’s *t* test or one/two-way ANOVA was used to compare two or more groups.

## Data availability

All relevant data are within the paper and its supporting information files.

## Supporting information

This article contains supporting information.

## Conflict of interest

The authors declare that they have no conflicts of interest with the contents of this article.
