# Supplementary material for: Calcium triggers calmodulin degradation to induce EGF receptor instability and overcome non-small cell lung cancer resistance to tyrosine kinase inhibitors
Source: J Biol Chem. 2025 May 28;301(7):110305. doi: 10.1016/j.jbc.2025.110305 (PMC12256327; doi:10.1016/j.jbc.2025.110305)
Supplement: Supporting Information [file mmc1.docx]

**Supplementary Figures S1-S4.**


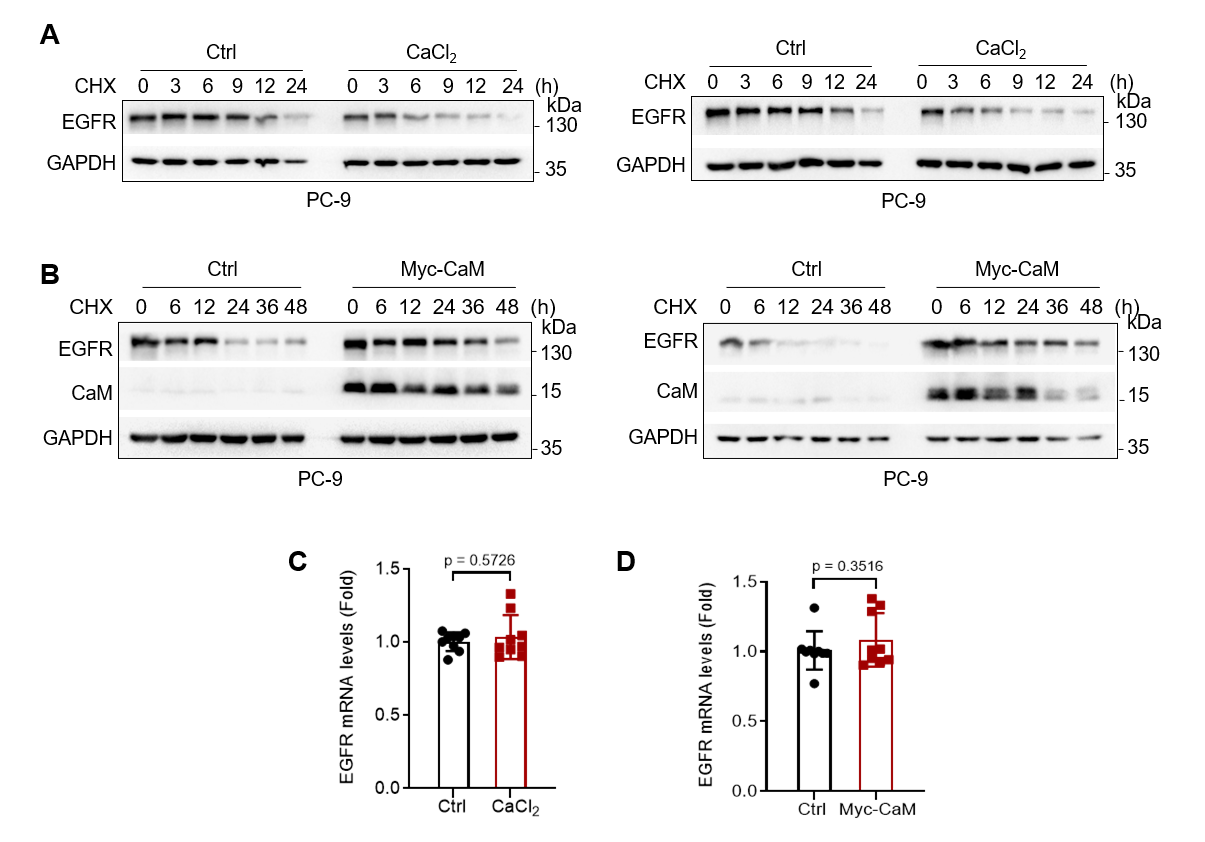
**Supplementary Figure S1**

**Supplementary Figure S1. Ca^2+^ or CaM can regulate EGFR protein stability, but has little effect on the steady-state mRNA levels of EGFR.** (A) The replicated experiments of Fig. 1L. PC-9 cells were treated with 1.5 mM CaCl_2_ for 24 h, and cells were then treated with 50 µg/ml CHX at indicated time prior to western blot analyses. (B) The replicated experiments of Fig. 1M. PC-9 cells stably expressing CaM or control were treated with 50 µg/ml CHX at indicated time prior to western blot analyses. (C) PC-9 cells were treated with or without 1.5 mM CaCl_2_ for 24 h, and the cells were then subjected to Q-PCR analyses. Data were presented as mean ± SD of three independent experiments performed in triplicates (t_16_ = 0.5761, unpaired Student’s *t*-test). (D) PC-9 cells stably expressing Myc-CaM or control were subjected to Q-PCR analyses. Data were presented as mean ± SD of three independent experiments performed in triplicates (t_16_ = 0.9594, unpaired Student’s *t*-test). Statistical analysis was perfomed using unpaired Student’s *t*-test.

**Supplementary Figure S2**


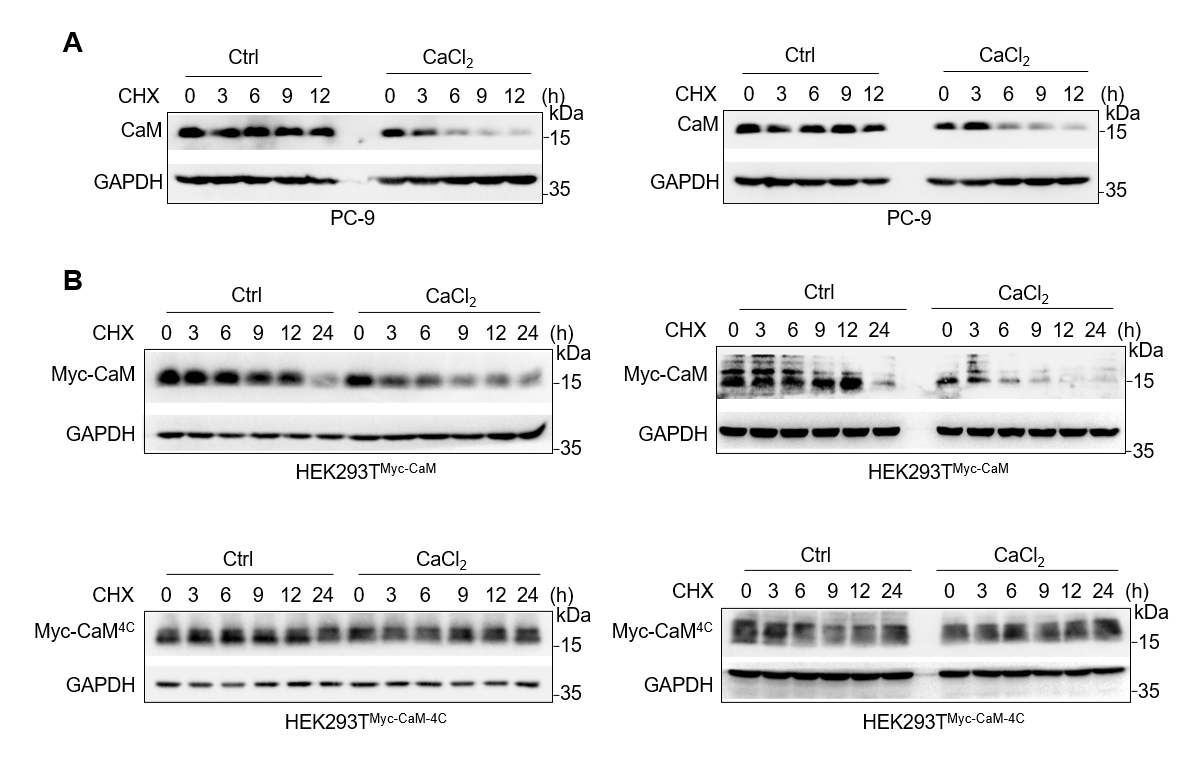


**Supplementary Figure S2. Ca^2+^ can promote CaM protein degradation, which is dependent on their binding.** (A) PC-9 cells were treated with 1.5 mM CaCl_2_ for 12 h, and cells were then treated with 50 µg/ml CHX at indicated time prior to western blot analyses. (B) HEK293T cells were transfected with Myc-CaM or Myc-CaM^4C^ expressing plasmids for 24 h. Cells were then treated with or without 1.5 mM CaCl_2_ for 24 h and 50 µg/ml CHX were added at indicated time prior to western blot analyses.


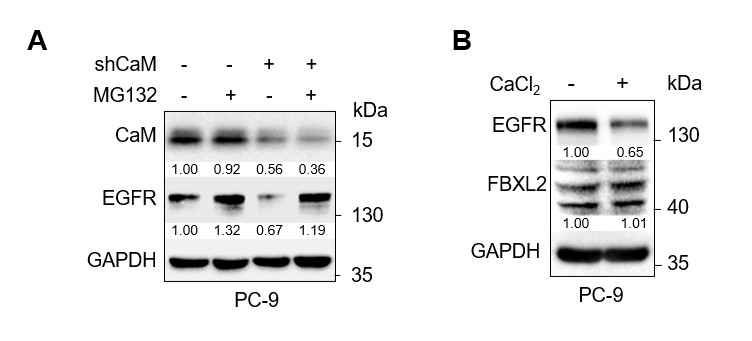
**Supplementary Figure S3**

**Supplementary Figure S3. Ca^2+^ has little effects on FBXL2 protein expression.** (A) PC-9 stable cells were treated with or without 20 µM MG132 for 6 h prior to western blot analyses. (B) PC-9 cells were treated with or without 1.5 mM CaCl_2_ for 24 h prior to western blot analyses. The protein bands in western blots were quantitated and normalized to GAPDH.


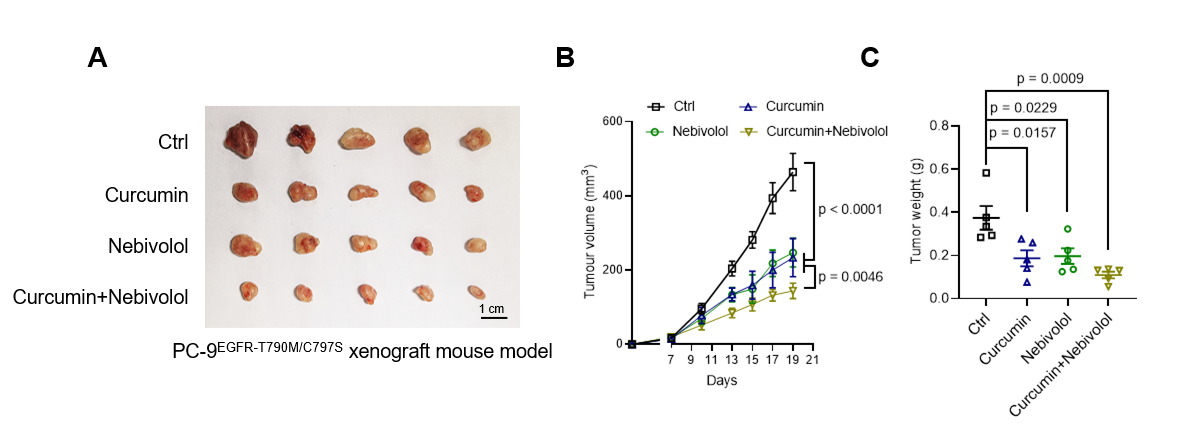
**Supplementary Figure S4**

**Supplementary Figure S4. The combination of curcumin and nebivolol significantly inhibits EGFR^T790M/C797S^-induced NSCLC growth.** (A-B) PC-9^EGFR-T790M/C797S^ cells were subjected to xenograft mouse model assay (n=5/group). Mice were administrated with curcumin, nebivolol alone or in a combination. The photos of tumor (A), tumor growth curves (B; time factor: F(6, 112) = 65.96, p < 0.0001, treatment factor: F(3, 112) = 31.89, p < 0.0001, and their interaction: F(18, 112) = 4.446, p < 0.0001, two-way ANOVA followed by uncorrected Fisher’s LSD test) and tumor weights (C; F(3, 16) = 8.488, p = 0.0013 using one-way ANOVA followed by Tukey’s multiple comparisons test) were shown. Data were presented as mean ± SEM of 5 biological replicates.
